# The Application of Metabolomics to Probiotic and Prebiotic Interventions in Human Clinical Studies

**DOI:** 10.3390/metabo10030120

**Published:** 2020-03-24

**Authors:** Thomas M. O’Connell

**Affiliations:** Department of Otolaryngology—Head & Neck Surgery, Indiana University School of Medicine, Indianapolis, IN 46202, USA; thoconne@iu.edu; Tel.: +1-919-621-1074

**Keywords:** microbiome, microbiota, probiotic, prebiotic, metabolomics

## Abstract

There is an ever-increasing appreciation for our gut microbiota that plays a crucial role in the maintenance of health, as well as the development of disease. Probiotics are live bacteria that are consumed to increase the population of beneficial bacteria and prebiotics are dietary substrates intended to promote the propagation of beneficial bacteria. In order to optimize the use of probiotics and prebiotics, a more complete biochemical understanding of the impact that these treatments have on the community and functioning of the gut microbiota is required. Nucleic acid sequencing methods can provide highly detailed information on the composition of the microbial communities but provide less information on the actual function. As bacteria impart much of their influence on the host through the production of metabolites, there is much to be learned by the application of metabolomics. The focus of this review is on the use of metabolomics in the study of probiotic and prebiotic treatments in the context of human clinical trials. Assessment of the current state of this research will help guide the design of future studies to further elucidate the biochemical mechanism by which probiotics and prebiotics function and pave the way toward more personalized applications.

## 1. Introduction

Research in the area of probiotic and prebiotic interventions has increased exponentially in recent decades. As defined by the International Scientific Association for Probiotics and Prebiotics, probiotics are live microorganisms that confer a health benefit on the host when administered in adequate amounts, whereas prebiotics are substrates that are selectively utilized by host microorganisms conferring a health benefit [1]. This notion of therapeutic modulation of the gut microbiota is hardly new, with the earliest investigations credited to the Nobel Prize-winning embryologist Elie Metchnikoff over a century ago. Metchnikoff posited that our microbial inhabitants released toxins that would lead to host damage [2]. In probably the earliest example of targeted probiotic therapy, he introduced *Bacillus bulgaricus* to individuals to mitigate the production of these harmful toxins. Another early pioneer in this area was Yale bacteriologist, Leo Rettger who reported in 1920 that the consumption of *Lactobacillus acidophilus* could successfully recolonize the human gut as a curative [3]. In this study, the co-administration of bacteria with certain carbohydrates is perhaps the earliest example of the combined use of probiotic and prebiotics, known now as synbiotics.

In the early 1990s, with the development of nucleic acid sequencing methods, the interest in the microbiome was re-invigorated. New methods including the analysis of ribosomal small subunit RNA gene probes, e.g., 16S ribosomal gene analysis, quickly found their way into the study of the intestinal environment. Computational methods enabled the determination of the microbial community with an unprecedented level of detail. However, beyond composition, it must be recognized that much of the impact of the bacterial community on the host is transmitted through the metabolites that are produced. Microbes produce or transform a wide range of circulating chemicals including amino acids, fatty acids, bile acids, hormones and vitamins [4,5].

The link between microbial community composition and metabolic activity is indirect and has been shown to be strongly influenced by functional redundancy and metabolic plasticity [6,7,8]. Functional redundancy indicates that some species can be interchangeable, while enacting similar functions. Thus, differences in microbial communities may not lead to differences in function. Metabolic plasticity reflects the ability of a given community to adapt to environmental changes by adjusting metabolic functioning. In order to better understand the ways in which probiotic and prebiotic interventions influence the host, analysis of the microbial community should also be accompanied by analysis of the function of these communities by examining the metabolome.

Experimental models on the use of probiotics and prebiotics have provided a detailed foundation on the physiological impact of these interventions. Murine studies have included investigations into diabetes, neurodevelopmental disorders, non-alcoholic steatohepatitis and more [9,10,11]. An especially noteworthy model approach involves the use of germ-free or gnotobiotic mice. The latter typically refers to mice that are hosts to a specifically defined microbiota. In one of the earliest examples of this type of study, fecal samples from obese patients were transplanted into germ-free mice which lead to an accumulation of body fat [12]. This study set the stage for further studies in these models to study the impact of probiotics and prebiotics. In the study by Martin et al., the ability of probiotics and prebiotics to impact the fecal metabolome was evaluated in three sets of mice with three distinct microbiota compositions [13]. The first set had a conventional (i.e., wild-type) microbiota. The second group was “conventionalized,” wherein germ-free mice were removed from their sterile incubators and place in normal cages. The last group were germ-free mice that were inoculated with human baby microbiota (HBM). All mice in the HBM group were supplemented with *Lactobacillus paracasei* and some of this group also received a prebiotic composed of galactooligosaccharides. Metabolomics analysis using ^1^H NMR revealed that the metabolome of the conventional and conventionalized quickly converged and were distinct from the HBM groups. Furthermore, the HBM group receiving only the probiotic was distinct from the group that received both the probiotic and prebiotic. This study leveraged the tremendous level of control available in mouse models to demonstrate how the fecal metabolome can provide unique information on nutrient-microbiota relationships. 

The ultimate goal of much of the research on probiotics and prebiotics is to aide in the design of more effective and personalized interventions to improve human health. Unfortunately, the exquisite level of control available in animal studies including rigorously controlled diets and environments, strictly defined probiotic and prebiotic dosing schedules and the opportunity for invasive sampling are not available in humans. The focus of this review is to evaluate the current state of the science in analyzing the effects of probiotics and prebiotics in the context of human clinical trials. These studies will be evaluated to understand the challenges in study design, analytical approaches and interpretation. Furthermore, the focus will only include those studies which employed a discovery-based metabolomics approach to more completely interrogate the action of prebiotics and probiotics on human health.

**Figure 1:**
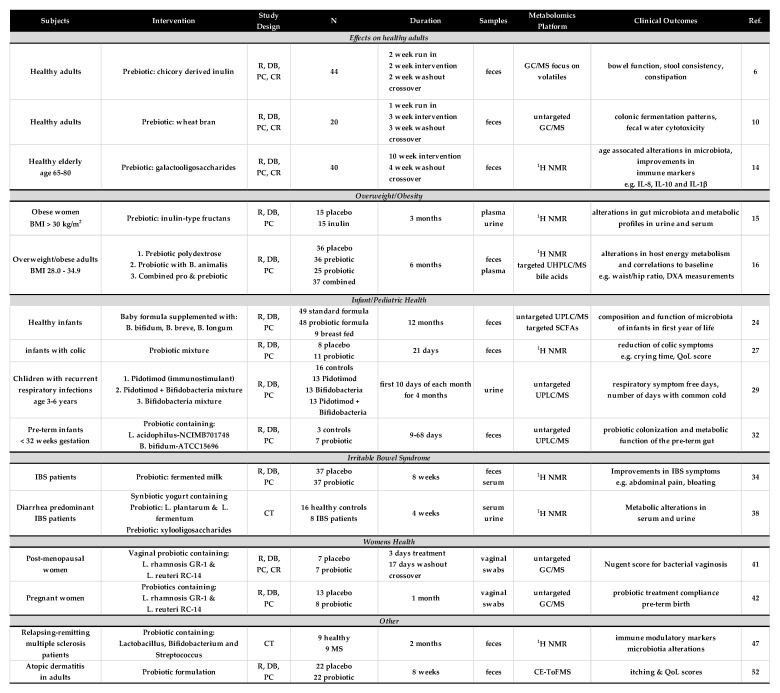
Description of human clinical studies of probiotic and prebiotic interventions that included the use of a discovery-based metabolomics analysis. Abbreviations: R, randomized; DB, double blind; PC, placebo controlled; CT, clinical trial.

The studies that met the focused criteria were found in PubMed using the search string (microbiome OR microbiota) AND (metabolomics OR “metabolite profiling” or “metabolic profiling”) AND (probiotics OR prebiotics). Careful review yielded 15 studies that reported results from some form of human clinical trial and included a discovery-based metabolomics approach. These studies were found to focus on five main areas of health and the results of these studies will be described below. Details of each of these studies are presented in Figure 1.

## 2. Effects on Healthy Subjects

One of the main questions regarding prebiotic and probiotic interventions is how much they can influence the community and function of a healthy gut. The study by Vandeputte et al. examined the effects of inulin-type fructans in a randomized, double-blind, placebo-controlled, crossover trial [14]. In this study, 44 healthy volunteers had a two-week run-in phase, followed by a four-week intervention, two-week washout and then crossover. 

The prebiotic intervention was composed of daily consumption of chicory derived inulin. Microbial community analysis was carried out using 16S rDNA gene sequencing. The results showed that there were significant alterations in the microbiota with inulin consumption. In particular, there was an increase in *Bifidobacteria* and *Anaerostipes*, along with a decrease in *Bilophilia*. The increase in *Bifidobacterium* was expected as the prebiotic concept actually emerged from the observation of selective stimulation of *Bifidobacterium* with inulin fermentation [1]. The metabolomics analysis utilized a GC/MS platform and was designed to only focus on the volatile organic compounds per the method of De Preter et al. [15]. This provided a significantly limited picture of the fecal metabolome, leaving out essentially all polar compounds including important organic acids such as lactate, succinate and formate. The cross-over analysis revealed one metabolite, dodecanal, which increased with inulin consumption. Dodecanal may be of dietary origin [16], but it may also be the result of altered intestinal lipid digestion [17].

The second study examined the prebiotic effects of wheat bran extracts (WBEs) on the microbial community and metabolome [18]. In addition to looking for alterations in the fecal microbiota and metabolome, this study examined the potential of this prebiotic intervention to impact risk markers for colorectal cancer. The study included 20 healthy subjects in a randomized, double-blind, placebo-controlled, crossover trial. Each subject consumed the wheat bran prebiotic or maltodextrin control for 3 weeks separated by a three-week washout period. Gut microbiota composition was measured by denaturing gradient gel electrophoresis, along with real-time PCR, and untargeted metabolomic analysis was carried out with a GC/MS platform. The colorectal cancer risk was evaluated using the comet assay to measure fecal water genotoxicity, as described by Wasson et al. [19].

The metabolomics analysis provided relative quantification on a total of 285 different metabolites with an average of 91 metabolites per sample. To provide more quantitative results of the short-chain fatty acid, 2-ethylbutyric acid, was used as an internal standard. Multivariate analysis using partial least squares discriminant analysis (PLS-DA) suggested that wheat bran intake was associated with higher levels of cycloalkenes, alcohols, and esters, but no individual metabolites met a threshold for significance.

Although the fecal water cytotoxicity and genotoxicity assays did not reveal a statistically significant difference between the prebiotic intervention and placebo, a PLS-DA showed that metabolite profiles were clustered based on the degree of cytotoxicity or genotoxicity of the samples. The analysis showed that acids, alcohols, and esters were associated with greater cytotoxicity, while cycloalkanes and a set of terpines including α-pinene, α- and β-phellandrene, limonene, 3-carene and camphene were associated with low toxicity. Terpines are secondary metabolites produced by plants and have been shown to have antioxidant and antimicrobial effects [20]. Another interesting metabolomic finding was a reduction in isovaleric acid with WBEs. This branched chain fatty acid is a marker protein fermentation which suggests that WBEs may alter the colonic fermentation pattern to be more saccharolytic rather than proteolytic.

During the aging process, alterations in the immune system have been observed and recent studies have shown that the microbiota and the microbial metabolome can impact certain immunological functions [21]. In the study by Vulevic et al., a prebiotic intervention using a galactooligoscaccharide (GOS) mixture was given to a cohort of elderly patients to observed the impact on the gut microbiota, fecal metabolome and immunological functions [22]. The study involved forty healthy patients between the ages of 65 and 80 in a randomized, double-blind, placebo-controlled, cross-over trial. The study design included ten-week interventions separated by a four-week wash-out. The GOS intervention was found to have immunomodulatory effects including alterations in NK cells and several cytokines, including an increase in Il-10 and a decrease in IL-1β. Principal changes in the microbiota were evaluated using fluorescence in situ hybridization. The main alteration included increases in *Bifidobacteria* and *Bacteroides*. The metabolomics analysis was carried out using ^1^H NMR and found that the increased levels of *Bifidobacterium* were correlated to an elevation in the fecal concentrations in lactate, glutamate, ornithine and caproic acid. As lactate is product of *Bifidobacerium* metabolism, this result supports the prebiotic simulation of that bacterium. The changes in glutamate and ornithine are likely associated with changes in the diet. Diet records did not indicate any differences in protein intake between the treatment groups, but when correlating *Bifidobacterium* levels with protein intake across all subjects, the levels of protein consumption were higher in those with higher *Bifidobacterium* levels. The metabolomics data in this study supported the use of prebiotics to alter both the level and function of Bifidobacterium and also demonstrated how metabolomics analysis can reveal additional insights into the effects of dietary patterns in the study.

## 3. Overweight/Obesity

The first associations between the gut microbiota and obesity were reported by Turnbaugh et al. in studies using fecal transplants from obese humans into germ-free mice [12]. When implanted with the “obese microbiota”, the mice experienced significantly greater increases in body fat than when implanted with the “lean microbiota”. Given the global health burden of obesity, there is a great interest in finding ways to manipulate the gut microbiota as a treatment for obesity. The search parameters found two studies where prebiotics or probiotics were evaluated in human studies of obesity using metabolomics.

In a study of obese women, the effects of inulin-type fructans (ITFs) was examined to evaluate the effects on gut microbial composition and systemic metabolism [23]. This double-blind, placebo-controlled intervention involved 30 obese women treated daily with an ITF supplement for three months. ^1^H NMR analysis of urine and serum revealed several interesting correlations between metabolite changes and gut bacteria. The levels of *Propionibacterium* and *Bacteroides vulgatus* were both decreased by the prebiotic treatment and correlated with decreased plasma levels of phosphatidylcholine and lactate. The levels of these bacteria and metabolites also appear to be associated with lower adiposity. An increase in the levels of *Collinsella*, a genus of *Actinobacteria* was observed in the prebiotic group and was correlated to the levels of urinary hippurate. In contrast to most studies which focused on the fecal metabolome, this study examined urine and serum and found several novel correlations between the gut microbiota and systemic metabolite levels.

Hibbert et al. reported a study involving both probiotic and prebiotic interventions in a randomized, double-blind, placebo-controlled trial of 134 overweight adults [24]. This study included four treatment groups (1) placebo (microcrystalline cellulose) (2) treatment with the prebiotic Litesse Ultra^®^ polydextrose (LU), (3) *Bifidobacterium animalis* subsp. *Lactis* 420^®^ (B420) and (4) LU + B420, with the intervention lasting six months. The fecal metabolome was analyzed by ^1^H NMR and serum bile acids were measured using UPLC/MS/MS as described by Han et al. [25]. The results showed that the gut microbiota was modified for all of the groups with the LU intervention showing a stronger effect than B420 alone. The abundance of the family *Christensenellaceae* was highly elevated in the groups treated with LU alone and in combination with B420. These levels were also positively correlated with the fecal branched chain fatty acids (BCFAs), isobutyric acid, isovaleric acid, 2-methylbutyric acid and 3-methyl-2-oxovalerate and the plasma bile acids, lithocholic acid and hyodeoxycholic acid. The BCFAs are typically generated by microbial fermentation of undigested proteins in the colon, but it has been shown that these metabolites can be derived from polydextrose consumption [26]. A recent study has shown that BCFAs affect human adipocyte lipid and glucose metabolism by inhibiting *de novo* lipogenesis [27]. In this study, the combined results of microbial community analysis and metabolomics provide new insights on how these interventions may affect host energy metabolism.

## 4. Infant and Pediatric Health

The composition of the infant gut microbiota has been shown to be dramatically influenced by dietary factors [28,29]. Clear differences in the trajectory of microbial community development have been shown between breastfed and formula-fed infants [30,31]. The study by Bazanella et al. was designed to evaluate the effects of a probiotic-supplemented formula intervention on the composition and function of the infant gut over the first year of life [32]. In this randomized, double-blind, placebo-controlled, clinical trial new-born infants were fed a standard whey-based formula supplemented with *Bifidobacterium bifidum, Bifidobacterium breve, Bifidobacterium longum* and *Bifidobacterium longum subspeces infantus* or a control formula with no supplements. Breastfed controls were also included in the analysis. The cohort was composed of 49 infants in the control group and 48 infants in the intervention. Diversity and composition of the fecal microbiota were determined by 16S rRNA sequencing and the fecal metabolome was analyzed by UPLC/MS. Infants in the intervention group showed decreased numbers of *Bacteroides* and *Blautia*, along with alterations in lipids and unknown metabolites at one month. Over the remainder of the study period, the microbiota composition and metabolome converged. Significant differences were observed between breast fed and formula fed infants with the major discriminating metabolites assigned to sterol lipids, glycerophospholipids and fatty acids. A targeted short-chain fatty acid analysis could also distinguish breast from formula fed infants with breast fed infants having lower proportions of propionate, butyrate, valerate and isovalerate, while formula fed infants had higher amounts of pyruvic acid and lactic acid.

Infantile colic is a condition experienced by somewhere between 10% and 40% of infants and is characterized by extended periods of inconsolable crying [33]. It is considered to be a gastrointestinal disorder, but the etiology remains unknown. Several studies have suggested that alterations in the infant gut microbiota could be contributing to this condition [34]. In the study by Baldassarre et al., a multi-strain probiotic supplement was used to treat infantile colic [35]. This randomized, double-blind, placebo-controlled, clinical trial involved daily oral probiotic treatment for 21 days. Microbial analysis with qPCR and metabolomics analysis with ^1^H NMR was carried out on 8 subjects from the placebo group and 11 from the probiotics group. The probiotic group experienced a significant decrease in minutes of crying per day at days 14 and 21 of the study. Interestingly, no significant changes in the composition of the microbiota were found over the course of the study, but 16 metabolites were found to be significantly different at day 21 between the placebo and probiotics groups. An interesting set of metabolites including the amino acids alanine, leucine, and isoleucine, along with the organic acids, 2-hydroxyisovalerate and 2-oxoisocaproate, were altered, suggesting a significant change in branched chain amino acid metabolism with the probiotic.

Recurrent respiratory tract infections (RRIs) are a common occurrence in preschool age children. Pidotimod is an immunostimulant that is used to treat RRIs in children. A recent metabolomics study of children with RRIs taking Pidotimod showed that the drug can partially restore the metabolic alterations induced by RRIs, but some of the microbiota derived metabolites remained altered [36]. In the follow-up study by Santamaria et al., a randomized, double-blind, placebo-controlled trial was conducted to determine if Pidotimod and/or a *Bifidobacteria* probiotic can reduce the morbidity of RRIs and alter the urinary metabolomics profiles in preschool children [37]. A cohort of children age 3–6 were randomly assigned to the four groups where subjects received treatments for the first 10 days of each month for four months. The primary clinical endpoint was the number of symptom free days. Children receiving Pidotimod either with or without probiotic had significantly more symptom free days. Metabolic profiles were measured on 25 patients using UPLC/MS. No significant differences in the metabolic profiles were observed between the placebo and probiotic group, but a large number were observed between the placebo and Pidotimod both with and without probiotic. Using a shared and unique structures plot they found that steroid hormones, metabolites derived from the vitamin B pathway and some amino acids were associated with the use of Pidotimod. At the extremes of this type of plot are the metabolites that can be related to an interactive effect of Pidotimod and the probiotic. These included methylhippuric acid, kynurenine, deoxycholic acid 3-glucuronide, and oxoglutaric acid. This set of metabolites are associated with a number of different aspects of gut microbial metabolism including tryptophan and bile acid metabolism. However, this pattern cannot be due specifically to the effects of the probiotic as these alterations were only observed when Pidotimod was administered with or without the probiotic. Given the clinical outcome, it is suspected that these metabolites are in some way, associated with increased immune function, but further work is required to elaborate.

Probiotics have been used in a number of studies of pre-term infants with much of the attention going toward the treatment of necrotizing enterocolitis and late onset sepsis [38]. For the former, evidence from more than 20 RCTs suggest a positive impact of probiotics [39]. In a study of pre-term infants, by Abdulkadir et al., 16S rRNA sequencing and LC/MS-based metabolomics were used to determine the effect of probiotics on pre-term infants in the neonatal intensive care unit (NICU) [40]. The probiotic consisted of *Lactobacillus acidophilus*-NCIMB701748 and *Bifidobacterium bifidum*-ATCC15696 (marketed as Infloran^®^). Fecal samples were collected before, during, and after probiotic intake from 7 patients and 3 time-matched controls. Samples were also collected following discharge from the NICU. The bacterial community profiling showed greater abundance of *Bifidobacterium* and *Lactobacillus* during the probiotic treatment compared to controls. After the supplementation stopped the *Lactobacillus* levels went down, but the *Bifidobacterium* concentrations remained high indicating successful colonization. The biggest difference in the metabolomics profiles was between subject post-discharge compared with those in the NICU. This would suggest that exposure to the new environment has a greater impact on these infants than the probiotic. Unfortunately, specific metabolite assignments were not made in this study.

## 5. Irritable Bowel Syndrome

Irritable bowel syndrome is the most common functional bowel disorder and is characterized by the association of abdominal pain or discomfort, with changes in stool frequency or consistency without a clear disease etiology [41]. In the study by Hong et al., targeted culture methods were combined with metabolomics to evaluate the effects of a probiotic yogurt drink on 74 IBS patients [42]. In this randomized, double-blind, placebo-controlled clinical trial subjects consumed the probiotic yogurt drink three times per day with meals over an eight-week period. The probiotic therapy yielded positive effects on the symptoms of the patients including pain, flatulence and defecations scores. The quantitative cell culture methods revealed that *Lactobacillus* spp. levels were significantly increased after the eight-week study, while the levels of *Bifidobacteria* did not change.

NMR-based metabolomics analyses were carried out on both serum and fecal samples. For both types of samples, no filtration or extraction methods were used so that the samples contained all of high molecular weight components including proteins and lipoproteins in serum and proteins, fiber and mucopolysaccharides in feces. Pattern recognition methods including principal components analysis were carried out on binned spectra. Analysis of the fecal samples suggested no significant differences between the placebo and probiotic treatment groups. The authors suggest that this is due to the confounding presence of unidentified metabolite peaks related to individual diets. The biggest effect found in the serum metabolomics analysis was between males and females. A well-known difference can be seen in the lipoprotein signatures of males and females in serum NMR spectra [43]. This led to separate analyses of the serum from each gender. No significant effects of the probiotics were found in the male subjects, but a weak difference in females was found using OPLS-DA. This analysis revealed increased lactate and reduced glucose and tyrosine levels in the female sera after the probiotic intervention. The increased lactate is sensible as the probiotic contains fermentative bacteria that produce lactate. Interestingly, the glucose and tyrosine levels after the probiotic treatment were very close to those found in the healthy subjects and significantly lower than before treatment. It is suggested that the reduced serum glucose levels may be the result of a probiotic rescue of impaired glycolysis in the subjects. A marked increase in serum tyrosine is observed in patients with liver disease [44] and so the decreased levels of tyrosine is posited to indicate an improvement in liver function. Hepatobiliary diseases are one of the most common comorbidities of IBD, therefore it is possible that the probiotic is having some corrective action on mild liver metabolic impairments [45].

This observation of lipoproteins enabled the detection of some potential sex related effects of the probiotics, but unfortunately the broad baseline features from these macromolecules interferes with the ability to quantitatively fit the NMR peaks. The spectral profiles were compared using integral binning which is less quantitative due to the effects of spectral overlap and interfering baseline components. This analysis approach was also used for the fecal samples. These added sources of variability may have limited ability to detect significant metabolite differences.

In another study of IBS, Noorbakhsh et al. used NMR-based metabolomics to investigate the metabolic impact of a synbiotic yogurt treatment on a cohort of diarrhea predominant IBS patients (IBS-D) [46]. This study examined the serum and urine metabolomes of 16 healthy and eight IBS-D patients before and after a four-week synbiotic yogurt intervention. The yogurt was cultured with *L. plantarum* and *L. fermentum* and contained xylooligosaccharides as a prebiotic. Fecal samples were cultured before and after the intervention. It was found that the number of viable fecal *Lactobacilli* from IBS-D patients was significantly lower at baseline than controls and increased significantly after the intervention. No differences were found in the control group before and after the intervention. Serum NMR showed several significant differences between the IBS-D and control groups before the intervention, including elevated levels of acetoacetate, myo-inositol and sarcosine, along with reduced levels of threonine and methionine. Comparing the serum metabolome of the IBS-D groups before and after the intervention revealed a decrease in acetone and an increase in the concentrations of choline, phenylalanine and the three branched chain amino acids, leucine, isoleucine and valine.

Several of the metabolites that distinguish between the healthy and IBS-D subjects point to alterations in one-carbon metabolism including serum methionine and sarcosine and urinary taurine. This led the authors to utilize an ELISA based assay to measure another key intermediate in the one-carbon pathway, homocysteine. The serum levels of homocysteine were found to be increased levels in the IBS-D group. After the intervention, the levels were reduced further suggesting that one mechanism by which the synbiotic treatment improves IBS-D symptoms may be through modulation of one-carbon metabolism.

## 6. Women’s Health/Pregnancy

The vaginal microbiota is part of a complex antimicrobial defense system that protects women against disease [47]. In the healthy state, the vaginal microbiota is dominated by the *Lactobacillus* genus [48]. It is thought that the lactate produced by these bacteria creates an acidic environment that restricts the growth of many pathogenic bacteria. The premenopausal vaginal microbiota has been shown to be highly dynamic compared with the post-menopausal state, likely due to the effects of hormone cycling. In the study by Bisanz et al., a detailed examination of the effects of a probiotic treatment on the post-menopausal vaginal microbiome was conducted [49]. In this study, seven subjects received vaginal administration of a probiotic composed of *L. rhamnosis* GR-1 and *L. reuteri* RC-14 and seven received a placebo treatment. The women were evaluated using the Nugent scoring system which is used to assess bacterial vaginosis (BV). All women in the study had intermediate scores indicative of a potential risk of transition to BV. The three-day treatment did not impact the Nugent score, but some changes in the microbial community were observed. Analysis with 16S rRNA sequencing revealed an increase in the relative abundance of *Lactobacillus*. An untargeted GC/MS analysis was conducted, but no differences were found between subjects on the probiotic and placebo. Obtaining a sufficient amount of sample for metabolomics was noted as a challenge using vaginal swabs and samples from only four women who demonstrated an increase in *Lactobacilli* were analyzed. A significant positive correlation between the lactate levels and the amount of lactate producing bacteria was found. The authors recognized this might seem to be an intuitive finding, but not that to their knowledge, this correlation had not yet been reported.

The next study of the use of probiotics on the vaginal microbiota, also conducted by the Reid Lab, evaluated the use of oral probiotics use in pregnant women in Rwanda [50]. The use of probiotics is not well established in developing counties like Rwanda, but the potential for treating and preventing may of the diseases that plague these countries like urogenital, diarrheal and respiratory diseases is very high [51,52,53]. In this randomized, blinded, placebo-controlled clinical trial, a total of 31 women completed the study (13 placebo, 18 probiotic), receiving daily oral supplements for one month. Vaginal swabs were used to collect samples for 16S rRNA sequencing and GC/MS based metabolomics.

The results showed the expected low bacterial diversity associated with a healthy vagina. No significant changes in the vaginal microbiota composition was observed between the placebo and probiotic treatment. Similarly, no significant changes were observed in the vaginal metabolome. Interestingly, women in the placebo group were significantly more likely to give birth preterm with three women in the placebo group compared with none in the probiotic group. Given the small size of this study, this intriguing finding will have to be validated in a larger cohort. Further analysis of the metabolomics data confirmed a previously observed set of biomarkers associated with bacterial diversity and bacterial vaginosis [54]. High levels of 2-hydroxyisovalerate and γ-hydroxybutyrate, both produced by *Lactobacillus crispatis* and BV-associated anaerobes were significantly higher in subjects with a high Nugent score.

## 7. Other Studies

The search criteria found studies in two areas for which there was only one example. The first was a study by Tankou et al. on the effects of a probiotic supplementation on relapsing remitting multiple sclerosis (MS) [55]. The gut microbiome has been implicated in several autoimmune disorders including MS, along with rheumatoid arthritis and inflammatory bowel disease [56,57,58,59]. In this study, nine MS patients and 13 healthy controls received a multi-strain probiotic containing *Lactobacillus, Bifidobacteria* and *Streptococcus* twice daily for 2 months. The study demonstrated that the probiotic increased the abundance of several taxa known to be depleted in MS such as *Lactobacillus*. Other taxa previously associated with dysbiosis in MS including *Akkermansia* and *Blautia* were found to be decreased with the probiotic further demonstrating the beneficial effects. Peripheral blood mononuclear cells (PBMC) were collected and gene expression studies showed a decrease in expression of a number of proinflammatory genes following probiotic administration. Correlation of the metabolomics with 16S rRNA sequencing data revealed a positive association between the *Lactobacillus* OUT 304,724 and the fecal hypoxanthine level. This metabolite also demonstrated a negative association with the decreased expression of the MS risk alleles HLA.DPB1 and HLD.DPA1 in PBMCs.

The use of a probiotic supplement in a study of atopic dermatitis in adults was reported by Matsumoto et al. [60]. Atopic dermatitis, also referred to as eczema, is a skin condition characterized by red and itchy skin. There is an increasing body of evidence that the gut microbiota plays a significant role in this disorder [61,62,63,64]. In this randomized, double-blind, placebo-controlled clinical study, 44 patients received a probiotic supplement containing *B. animalis* subsp *lactis* LKM512 or placebo daily for 8 weeks. The probiotic provided a significant benefit to the patients as evaluated by reduced itching and dermatology-specific quality of life scores. Real-time PCR confirmed that the patients on probiotics had increased fecal levels of *B. animalis* subsp *lactis* LKM512. The fecal metabolomics from three patients who experienced dramatic improvement in their symptoms with the probiotics revealed a significant increase in kynurenic acid. Increased levels of this metabolite were also shown to be negatively associated with itching symptoms.

A previous study by this group on a similar cohort of adults with atopic dermatis found that consumption of a probiotic LKM512 yogurt for only four weeks lead a similar improvement in symptoms [65]. In this study, they performed HPLC analyses of fecal polyamines and short-chain fatty acids. These metabolites were targeted due to their association with the maintenance and maturation of intestinal mucosal barrier function which has been shown to weakened in AD patients [66,67,68,69,70,71,72]. It is interesting to note that none of these metabolites were found to be significantly altered when profiled in the untargeted manner using the LC/MS method.

## 8. Discussion and Conclusions

With the increasing interest in the use of prebiotic and probiotic interventions for a wide range of diseases, it is becoming more and more important to understand the detailed biochemical mechanisms through which they function. As discussed earlier, analysis of the microbial community structure using gene sequencing methods may not provide information on the actual functioning of the bacteria. Despite a plethora of pre-clinical studies, there have been relatively few studies of the use of probiotics and prebiotics in the context of human clinical trials that have taken advantage of advanced metabolomics approaches. The examples presented here cover a range of features including pathological condition, cohort size, dosing frequency and duration. The intent of this review was to evaluate how these studies were designed and analyzed and consider ways in which future studies could be optimized.

A review of the study designs of the clinical trials described here shows one important variable that was generally not interrogated. As stated in the definition of probiotics and prebiotics, these therapies need to be “administered in adequate amounts” in order to provide the beneficial effects to the host. In the studies described, the dose, frequency and duration of the interventions were set at the beginning of the study and no clear indications of how these parameters were derived was given. Defining the dosing for a pharmaceutical involves a dose–response study with the guiding principal of “start low, go slow”. As probiotics and prebiotics are generally regarded as safe, such dose–response studies would hold far less risk than with a pharmaceutical. The obvious challenge with dose-range studies is that they will require larger numbers of patients to evaluate the effects of dosing, frequency and duration, but at some point, these types of studies will have to be carried out. The most practical applications might be in well-defined cohorts, e.g., patients with a specified pathology. In this way, the outcome phenotype should be strong enough that smaller cohorts may still have the statistical power to determine the effective dosing strategy.

As with any metabolomics analysis, there are a number of analytical sources of variation that need to be considered. The range of analytical platforms used in the studies discussed here are representative of the rest of the metabolomics field. The advantages and disadvantages of these platforms has been extensively reviewed and a summary of the different platforms used in fecal metabolome analysis is presented in the review by Karu et al. [73,74]. Given the strengths and weaknesses of each platform, especially in terms of sensitivity, quantitation and reproducibility, the use of multiple platforms would be highly advantageous. Examples of this were seen in several of the studies presented where global, untargeted analyses were combined with targeted metabolomics to provide more quantitative measurements of important metabolites such as short-chain fatty acids that may be missed by the primary untargeted approach.

A challenge that is specific to metabolomics of fecal samples is the stabilization of the fecal metabolome. In studying the serum metabolome, the protocol requires that the serum be quickly separated from the metabolically active red blood cells. Similarly, the fecal metabolites need to be protected from continued metabolism by the biologically active bacteria. Rapid freezing at −4 °C or lower as well as storage in 95% ethanol have been shown to have provide sufficient protection against continued metabolism [75]. In human studies, especially when the samples are collected at home and then delivered to the clinic, there will almost certainly be some alterations in the metabolome occurring during that time period. This additional source of variation may limit the observation of significant results where many studies will have small effect sizes. In the future, more rapid and robust means of stabilization that do not interfere with the metabolomic profiling are needed.

Another source of variation in fecal samples is the water content. Fecal samples typically contain 60%–85% water, depending upon fiber intake [76]. This variability is less than typically found in urine samples but is still significant when searching for potentially small metabolite changes. As with urine samples, there are data processing methods that will help minimize these effects. The use of “constant sum normalization,” where the total spectral area for NMR and total ion count for MS is set to a selected value, is often used. Probabilistic quotient normalization is another post-processing normalization method that has been used to account for variability in fecal composition [77]. Alternatively, data can be collected on lyophilized samples, but this can affect metabolite composition. Interestingly, in most of the studies discussed, little detail on the application of normalization methods was included, which may have reduced the information content of the studies.

The studies reviewed here demonstrate the potential of metabolomics to contribute to the understanding of how prebiotics and probiotics function in humans. Continued work on the development of standardized protocols for fecal metabolomics will help minimize both the biological and analytical sources of variation. These developments, along with the use of multi-platform approaches, will provide more detailed and comprehensive metabolomics data. Combining this data with advanced microbial sequencing analyses will help elucidate the detailed biochemical mechanisms by which probiotics and prebiotics can be optimally applied to improve human health.

## References

[1] Gibson G.R., Hutkins R., Sanders M.E., Prescott S.L., Reimer R.A., Salminen S.J., Scott K., Stanton C., Swanson K.S., Cani P.D. (2017). Expert consensus document: The International Scientific Association for Probiotics and Prebiotics (ISAPP) consensus statement on the definition and scope of prebiotics. Nat. Rev. Gastroenterol. Hepatol..

[2] Elie M. (1908). The Prolongation of Life: Optimistic Studies.

[3] Cheplin H.A., Rettger L.F. (1920). Studies on the transformation of the intestinal flora, with special reference to the implantation of bacillus acidophilus: II. Feeding experiments on man. Proc. Natl. Acad. Sci. USA.

[4] Clarke G., Stilling R.M., Kennedy P.J., Stanton C., Cryan J.F., Dinan T.G. (2014). Minireview: Gut microbiota: The neglected endocrine organ. Mol. Endocrinol..

[5] Pedersen H.K., Gudmundsdottir V., Nielsen H.B., Hyotylainen T., Nielsen T., Jensen B.A., Forslund K., Hildebrand F., Prifti E., Falony G. (2016). Human gut microbes impact host serum metabolome and insulin sensitivity. Nature.

[6] Allison S.D., Martiny J.B. (2008). Colloquium paper: Resistance, resilience, and redundancy in microbial communities. Proc. Natl. Acad. Sci. USA.

[7] Comte J., Fauteux L., Del Giorgio P.A. (2013). Links between metabolic plasticity and functional redundancy in freshwater bacterioplankton communities. Front. Microbiol..

[8] Moya A., Ferrer M. (2016). Functional Redundancy-Induced Stability of Gut Microbiota Subjected to Disturbance. Trends Microbiol..

[9] Ma J., Zhou Q., Li H. (2017). Gut microbiota and nonalcoholic fatty liver disease: Insights on mechanisms and therapy. Nutrients.

[10] Mangiola F., Ianiro G., Franceschi F., Fagiuoli S., Gasbarrini G., Gasbarrini A. (2016). Gut microbiota in autism and mood disorders. World J. Gastroenterol..

[11] Vallianou N.G., Stratigou T., Tsagarakis S. (2018). Microbiome and diabetes: Where are we now?. Diabetes Res. Clin. Pract..

[12] Turnbaugh P.J., Ley R.E., Mahowald M.A., Magrini V., Mardis E.R., Gordon J.I. (2006). An obesity-associated gut microbiome with increased capacity for energy harvest. Nature.

[13] Martin F.P., Sprenger N., Montoliu I., Rezzi S., Kochhar S., Nicholson J.K. (2010). Dietary modulation of gut functional ecology studied by fecal metabonomics. J. Proteome Res..

[14] Vandeputte D., Falony G., Vieira-Silva S., Wang J., Sailer M., Theis S., Verbeke K., Raes J. (2017). Prebiotic inulin-type fructans induce specific changes in the human gut microbiota. Gut.

[15] De Preter V., van Staeyen G., Esser D., Rutgeerts P., Verbeke K. (2009). Development of a screening method to determine the pattern of fermentation metabolites in faecal samples using on-line purge-and-trap gas chromatographic-mass spectrometric analysis. J. Chromatogr. A.

[16] Burdock G.A. (1996). Encyclopedia of Food and Color Additives.

[17] Lairon D. (1996). Dietary fibres: Effects on lipid metabolism and mechanisms of action. Eur. J. Clin. Nutr..

[18] Windey K., de Preter V., Huys G., Broekaert W.F., Delcour J.A., Louat T., Herman J., Verbeke K. (2015). Wheat bran extract alters colonic fermentation and microbial composition, but does not affect faecal water toxicity: A randomised controlled trial in healthy subjects. Br. J. Nutr..

[19] Wasson G.R., McKelvey-Martin V.J., Downes C.S. (2008). The use of the comet assay in the study of human nutrition and cancer. Mutagenesis.

[20] Lang G., Buchbauer G. (2012). A review on recent research results (2008–2010) on essential oils as antimicrobials and antifungals. A review. Flavor Fragr. J..

[21] Chen V.L., Kasper D.L. (2014). Interactions between the intestinal microbiota and innate lymphoid cells. Gut Microbes.

[22] Vulevic J., Juric A., Walton G.E., Claus S.P., Tzortzis G., Toward R.E., Gibson G.R. (2015). Influence of galacto-oligosaccharide mixture (B-GOS) on gut microbiota, immune parameters and metabonomics in elderly persons. Br. J. Nutr..

[23] Dewulf E.M., Cani P.D., Claus S.P., Fuentes S., Puylaert P.G., Neyrinck A.M., Bindels L.B., de Vos W.M., Gibson G.R., Thissen J.P. (2013). Insight into the prebiotic concept: Lessons from an exploratory, double blind intervention study with inulin-type fructans in obese women. Gut.

[24] Hibberd A.A., Yde C.C., Ziegler M.L., Honore A.H., Saarinen M.T., Lahtinen S., Stahl B., Jensen H.M., Stenman L.K. (2019). Probiotic or synbiotic alters the gut microbiota and metabolism in a randomised controlled trial of weight management in overweight adults. Benef. Microbes.

[25] Han J., Liu Y., Wang R., Yang J., Ling V., Borchers C.H. (2015). Metabolic profiling of bile acids in human and mouse blood by LC-MS/MS in combination with phospholipid-depletion solid-phase extraction. Anal. Chem..

[26] Jie Z., Bang-Yao L., Ming-Jie X., Hai-Wei L., Zu-Kang Z., Ting-Song W., Craig S.A. (2000). Studies on the effects of polydextrose intake on physiologic functions in Chinese people. Am. J. Clin. Nutr..

[27] Heimann E., Nyman M., Palbrink A.K., Lindkvist-Petersson K., Degerman E. (2016). Branched short-chain fatty acids modulate glucose and lipid metabolism in primary adipocytes. Adipocyte.

[28] Koenig J.E., Spor A., Scalfone N., Fricker A.D., Stombaugh J., Knight R., Angenent L.T., Ley R.E. (2011). Succession of microbial consortia in the developing infant gut microbiome. Proc. Natl. Acad. Sci. USA.

[29] Backhed F., Roswall J., Peng Y., Feng Q., Jia H., Kovatcheva-Datchary P., Li Y., Xia Y., Xie H., Zhong H. (2015). Dynamics and stabilization of the human gut microbiome during the first year of life. Cell Host Microbe.

[30] Guaraldi F., Salvatori G. (2012). Effect of breast and formula feeding on gut microbiota shaping in newborns. Front. Cell. Infect Microbiol..

[31] Tannock G.W., Lawley B., Munro K., Gowri Pathmanathan S., Zhou S.J., Makrides M., Gibson R.A., Sullivan T., Prosser C.G., Lowry D. (2013). Comparison of the compositions of the stool microbiotas of infants fed goat milk formula, cow milk-based formula, or breast milk. Appl. Environ. Microbiol..

[32] Bazanella M., Maier T.V., Clavel T., Lagkouvardos I., Lucio M., Maldonado-Gomez M.X., Autran C., Walter J., Bode L., Schmitt-Kopplin P. (2017). Randomized controlled trial on the impact of early-life intervention with bifidobacteria on the healthy infant fecal microbiota and metabolome. Am. J. Clin. Nutr..

[33] Lucassen P.L., Assendelft W.J. (2001). Systematic review of treatments for infant colic. Pediatrics.

[34] Camilleri M., Park S.Y., Scarpato E., Staiano A. (2017). Exploring hypotheses and rationale for causes of infantile colic. Neurogastroenterol. Motil..

[35] Baldassarre M.E., Di Mauro A., Tafuri S., Rizzo V., Gallone M.S., Mastromarino P., Capobianco D., Laghi L., Zhu C., Capozza M. (2018). Effectiveness and safety of a probiotic-mixture for the treatment of infantile colic: A double-blind, randomized, placebo-controlled clinical trial with fecal real-time pcr and nmr-based metabolomics Analysis. Nutrients.

[36] Bozzetto S., Pirillo P., Carraro S., Berardi M., Cesca L., Stocchero M., Giordano G., Zanconato S., Baraldi E. (2017). Metabolomic profile of children with recurrent respiratory infections. Pharmacol. Res..

[37] Santamaria F., Montella S., Stocchero M., Pirillo P., Bozzetto S., Giordano G., Poeta M., Baraldi E. (2019). Effects of pidotimod and bifidobacteria mixture on clinical symptoms and urinary metabolomic profile of children with recurrent respiratory infections: A randomized placebo-controlled trial. Pulm. Pharmacol. Ther..

[38] Gaul J. (2008). Probiotics for the prevention of necrotizing enterocolitis. Neonatal. Netw..

[39] Ofek Shlomai N., Deshpande G., Rao S., Patole S. (2014). Probiotics for preterm neonates: What will it take to change clinical practice?. Neonatology.

[40] Abdulkadir B., Nelson A., Skeath T., Marrs E.C., Perry J.D., Cummings S.P., Embleton N.D., Berrington J.E., Stewart C.J. (2016). Routine use of probiotics in preterm infants: Longitudinal impact on the microbiome and metabolome. Neonatology.

[41] Canavan C., West J., Card T. (2014). The epidemiology of irritable bowel syndrome. Clin. Epidemiol..

[42] Hong Y.S., Hong K.S., Park M.H., Ahn Y.T., Lee J.H., Huh C.S., Lee J., Kim I.K., Hwang G.S., Kim J.S. (2011). Metabonomic understanding of probiotic effects in humans with irritable bowel syndrome. J. Clin. Gastroenterol..

[43] Kochhar S., Jacobs D.M., Ramadan Z., Berruex F., Fuerholz A., Fay L.B. (2006). Probing gender-specific metabolism differences in humans by nuclear magnetic resonance-based metabonomics. Anal. Biochem..

[44] Levine R.J., Conn H.O. (1967). Tyrosine metabolism in patients with liver disease. J. Clin. Investig..

[45] Greenstein A.J., Janowitz H.D., Sachar D.B. (1976). The extra-intestinal complications of Crohn’s disease and ulcerative colitis: A study of 700 patients. Medicine.

[46] Noorbakhsh H., Yavarmanesh M., Mortazavi S.A., Adibi P., Moazzami A.A. (2019). Metabolomics analysis revealed metabolic changes in patients with diarrhea-predominant irritable bowel syndrome and metabolic responses to a synbiotic yogurt intervention. Eur. J. Nutr..

[47] Sobel J.D. (1999). Is there a protective role for vaginal flora?. Curr. Infect Dis. Rep..

[48] Yamamoto T., Zhou X., Williams C.J., Hochwalt A., Forney L.J. (2009). Bacterial populations in the vaginas of healthy adolescent women. J. Pediatr. Adolesc. Gynecol..

[49] Bisanz J.E., Seney S., McMillan A., Vongsa R., Koenig D., Wong L., Dvoracek B., Gloor G.B., Sumarah M., Ford B. (2014). A systems biology approach investigating the effect of probiotics on the vaginal microbiome and host responses in a double blind, placebo-controlled clinical trial of post-menopausal women. PLoS ONE.

[50] McMillan A., Rulisa S., Gloor G.B., Macklaim J.M., Sumarah M., Reid G. (2018). Pilot assessment of probiotics for pregnant women in Rwanda. PLoS ONE.

[51] Reid G., Kumar H., Khan A.I., Rautava S., Tobin J., Salminen S. (2016). The case in favour of probiotics before, during and after pregnancy: Insights from the first 1500 days. Benef. Microbes.

[52] Smid M.C., Stringer E.M., Stringer J.S. (2016). A Worldwide epidemic: The problem and challenges of preterm birth in low- and middle-income countries. Am. J. Perinatol..

[53] Vos T., Barber R.M., Bell B., Bertozzi-Villa A., Biryukov S., Bolliger I., Charlson F., Davis A., Degenhardt L., Dicker D. (2015). Global, regional and national incidence, prevalence and years live with disability for 301 acute and chronic diseases and injuries in 188 countries, 1990–2013: A systematic analysis for the Global Burden of Disease Study. Lancet.

[54] McMillan A., Rulisa S., Sumarah M., Macklaim J.M., Renaud J., Bisanz J.E., Gloor G.B., Reid G. (2015). A multi-platform metabolomics approach identifies highly specific biomarkers of bacterial diversity in the vagina of pregnant and non-pregnant women. Sci. Rep..

[55] Tankou S.K., Regev K., Healy B.C., Tjon E., Laghi L., Cox L.M., Kivisakk P., Pierre I.V., Hrishikesh L., Gandhi R. (2018). A probiotic modulates the microbiome and immunity in multiple sclerosis. Ann. Neurol..

[56] Jangi S., Gandhi R., Cox L.M., Li N., von Glehn F., Yan R., Patel B., Mazzola M.A., Liu S., Glanz B.L. (2016). Alterations of the human gut microbiome in multiple sclerosis. Nat. Commun..

[57] Zhang X., Zhang D., Jia H., Feng Q., Wang D., Liang D., Wu X., Li J., Tang L., Li Y. (2015). The oral and gut microbiomes are perturbed in rheumatoid arthritis and partly normalized after treatment. Nat. Med..

[58] Miyake S., Kim S., Suda W., Oshima K., Nakamura M., Matsuoka T., Chihara N., Tomita A., Sato W., Kim S.W. (2015). Dysbiosis in the gut microbiota of patients with multiple sclerosis, with a striking depletion of species belonging to clostridia XIVa and IV clusters. PLoS ONE.

[59] Chen J., Chia N., Kalari K.R., Yao J.Z., Novotna M., Paz Soldan M.M., Luckey D.H., Marietta E.V., Jeraldo P.R., Chen X. (2016). Multiple sclerosis patients have a distinct gut microbiota compared to healthy controls. Sci. Rep..

[60] Matsumoto M., Ebata T., Hirooka J., Hosoya R., Inoue N., Itami S., Tsuji K., Yaginuma T., Muramatsu K., Nakamura A. (2014). Antipruritic effects of the probiotic strain LKM512 in adults with atopic dermatitis. Ann. Allergy Asthma Immunol..

[61] Bjorksten B., Naaber P., Sepp E., Mikelsaar M. (1999). The intestinal microflora in allergic Estonian and Swedish 2-year-old children. Clin. Exp. Allergy.

[62] Kalliomaki M., Kirjavainen P., Eerola E., Kero P., Salminen S., Isolauri E. (2001). Distinct patterns of neonatal gut microflora in infants in whom atopy was and was not developing. J. Allergy Clin. Immunol..

[63] Penders J., Thijs C., van den Brandt P.A., Kummeling I., Snijders B., Stelma F., Adams H., van Ree R., Stobberingh E.E. (2007). Gut microbiota composition and development of atopic manifestations in infancy: The KOALA Birth Cohort Study. Gut.

[64] Abrahamsson T.R., Jakobsson H.E., Andersson A.F., Bjorksten B., Engstrand L., Jenmalm M.C. (2012). Low diversity of the gut microbiota in infants with atopic eczema. J. Allergy Clin. Immunol..

[65] Matsumoto M., Aranami A., Ishige A., Watanabe K., Benno Y. (2007). LKM512 yogurt consumption improves the intestinal environment and induces the T-helper type 1 cytokine in adult patients with intractable atopic dermatitis. Clin. Exp. Allergy.

[66] Dufour C., Dandrifosse G., Forget P., Vermesse F., Romain N., Lepoint P. (1988). Spermine and spermidine induce intestinal maturation in the rat. Gastroenterology.

[67] Wang J.Y., McCormack S.A., Viar M.J., Johnson L.R. (1991). Stimulation of proximal small intestinal mucosal growth by luminal polyamines. Am. J. Physiol..

[68] Buts J.P., de Keyser N., Kolanowski J., Sokal E., Van Hoof F. (1993). Maturation of villus and crypt cell functions in rat small intestine. Role of dietary polyamines. Dig. Dis. Sci..

[69] Deloyer P., Peulen O., Dandrifosse G. (2001). Dietary polyamines and non-neoplastic growth and disease. Eur. J. Gastroenterol. Hepatol..

[70] Kanauchi O., Iwanaga T., Mitsuyama K., Saiki T., Tsuruta O., Noguchi K., Toyonaga A. (1999). Butyrate from bacterial fermentation of germinated barley foodstuff preserves intestinal barrier function in experimental colitis in the rat model. J. Gastroenterol. Hepatol..

[71] Mariadason J.M., Barkla D.H., Gibson P.R. (1997). Effect of short-chain fatty acids on paracellular permeability in Caco-2 intestinal epithelium model. Am. J. Physiol..

[72] Venkatraman A., Ramakrishna B.S., Pulimood A.B. (1999). Butyrate hastens restoration of barrier function after thermal and detergent injury to rat distal colon In Vitro. Scand. J. Gastroenterol..

[73] Pinu F.R., Goldansaz S.A., Jaine J. (2019). Translational metabolomics: Current challenges and future opportunities. Metabolites.

[74] Karu N., Deng L., Slae M., Guo A.C., Sajed T., Huynh H., Wine E., Wishart D.S. (2018). A review on human fecal metabolomics: Methods, applications and the human fecal metabolome database. Anal. Chim. Acta.

[75] Gratton J., Phetcharaburanin J., Mullish B.H., Williams H.R., Thursz M., Nicholson J.K., Holmes E., Marchesi J.R., Li J.V. (2016). Optimized sample handling strategy for metabolic profiling of human feces. Anal. Chem..

[76] Bliss D.Z., Savik K., Jung H., Jensen L., LeMoine M., Lowry A. (1999). Comparison of subjective classification of stool consistency and stool water content. J. Wound Ostomy Cont. Nurs.

[77] Dieterle F., Ross A., Schlotterbeck G., Senn H. (2006). Probabilistic quotient normalization as robust method to account for dilution of complex biological mixtures. Application in 1H NMR metabonomics. Anal. Chem..

